# Intracellular Major Histocompatibility Complex Class II and C-X-C Motif Chemokine Ligand 10-Expressing Neutrophils Indicate the State of Anti-Tumor Activity Induced by *Bacillus Calmette–Guérin*

**DOI:** 10.3390/biomedicines11113062

**Published:** 2023-11-15

**Authors:** Yuji Takeda, Tomoyuki Kato, Saima Sabrina, Sei Naito, Hiromi Ito, Naoto Emi, Yuya Kuboki, Yuki Takai, Hiroki Fukuhara, Masaki Ushijima, Takafumi Narisawa, Mayu Yagi, Hidenori Kanno, Toshihiko Sakurai, Hayato Nishida, Akemi Araki, Yoshitaka Shimotai, Mikako Nagashima, Yusuke Nouchi, Shinichi Saitoh, Hidetoshi Nara, Norihiko Tsuchiya, Hironobu Asao

**Affiliations:** 1 Department of Immunology, Faculty of Medicine, Yamagata University, Yamagata 990-9585, Japan; shefa_610@yahoo.co.in (S.S.); m191238@st.yamagata-u.ac.jp (N.E.); mikako-nagashima@med.id.yamagata-u.ac.jp (M.N.); yusuke.n97@gmail.com (Y.N.); s-saitoh@med.id.yamagata-u.ac.jp (S.S.); asao-h@med.id.yamagata-u.ac.jp (H.A.); 2 Department of Urology, Faculty of Medicine, Yamagata University, Yamagata 990-9585, Japan; kato-t@med.id.yamagata-u.ac.jp (T.K.); seinaito@med.id.yamagata-u.ac.jp (S.N.); ito.hiromi@med.id.yamagata-u.ac.jp (H.I.); yuyakbk@gmail.com (Y.K.); t0a8_r1o2@yahoo.co.jp (Y.T.); hiroki_fukuhara@yahoo.co.jp (H.F.); uroushi@gmail.com (M.U.); tnari_0623@yahoo.co.jp (T.N.); mayu11ism05@gmail.com (M.Y.); ocean_ape@yahoo.co.jp (H.K.); tsakurai@med.id.yamagata-u.ac.jp (T.S.); hnishida331@yahoo.co.jp (H.N.); ntsuchiya@med.id.yamagata-u.ac.jp (N.T.); 3 Department of Infectious Diseases, Faculty of Medicine, Yamagata University, Yamagata 990-9585, Japan; yoshimo@med.id.yamagata-u.ac.jp; 4 Department of Biological Sciences, Faculty of Science and Engineering, Ishinomaki Senshu University, Miyagi 986-8580, Japan; h-nara@isenshu-u.ac.jp

**Keywords:** *Bacillus Calmette–Guérin* (BCG), bladder cancer, C-X-C motif chemokine ligand 10 (CXCL10), major histocompatibility complex (MHC) class II, myeloid-derived suppressor cells (MDSCs), neutrophils

## Abstract

(1) Background: Inflammatory responses induce the formation of both anti-tumor and pro-tumor neutrophils known as myeloid-derived suppressor cells (MDSCs). Intermittent intravesical infusion of *Bacillus Calmette–Guérin* (BCG) is an established cancer immunotherapy for non-muscle-invasive bladder cancer (NMIBC). However, the types of neutrophils induced via the inflammatory response to both tumor-bearing and BCG remain unclear. (2) Methods: We therefore analyzed neutrophil dynamics in the peripheral blood and urine of patients with NMIBC who received BCG therapy. Further, we analyzed the effects of BCG in a mouse intraperitoneal tumor model. (3) Results: BCG therapy induced the formation of CXCL10 and MHC class II-positive neutrophils in the urine of patients with NMIBC but did not induce MDSC formation. CXCL10- and MHC class II-expressing neutrophils were detected in peritoneal exudate cells formed after BCG administration. Partial neutrophil depletion using an anti-Ly6G antibody suppressed the upregulation of CXCL10 and MHC class II in neutrophils and reversed the anti-tumor activity of BCG in mouse models. (4) Conclusions: These results indicated that intracellular MHC class II- and CXCL10-expressing neutrophils indicate the state of anti-tumor activity induced via BCG. The status of neutrophils in mixed inflammation of immunosuppressive and anti-tumor responses may therefore be useful for evaluating immunological systemic conditions.

## 1. Introduction

Neutrophils are essential for the defense against infection and play key roles in the regulation of adaptive immune responses and chronic inflammation. Neutrophil diversity is thought to be caused by differences in the combined actions of several cytokines and other factors during the differentiation and maturation processes of neutrophils [1]. Recent studies have reported the classification of neutrophils into pro- and anti-tumor neutrophils [2,3].

Innate immune training during granulopoiesis promotes anti-tumor activity [4]. In contrast, while myeloid-derived suppressor cells (MDSCs) exhibit a negative aspect to anti-tumor immunity, they play an important role when physiological immunosuppression is required, such as during pregnancy [5,6]. The modulation of immune training and MDSCs allow for their application in the treatment of various diseases.

*Mycobacterium bovis*-derived *Bacillus Calmette–Guérin* (BCG) is well-known historically and empirically to mediate an artificial intervention to neutrophil diversity. BCG induces innate immune training [4,7] and triggers the accumulation of MDSCs [8,9]. One of the most effective treatments using BCG involves its use in the treatment of bladder cancer. Intravesical infusions of BCG have been used to treat non-muscle-invasive bladder cancer (NMIBC) since 1976, and continues to stand at the forefront of therapeutic options for this malignancy [10]. Intravesical instillation of BCG is usually administered once a week and repeated at least five times. Several studies have shown that neutrophils are required for effective BCG therapy [10,11,12]. However, the type of neutrophils that are produced during BCG intravesical infusion therapy remain unknown.

Neutrophil diversity is complex and dynamic during inflammatory responses [13]. Furthermore, the differentiation processes of neutrophils, monocytes, and MDSCs are sequential and mutually plastic [14,15,16]. Similarly, their morphologies possess plasticity, making it difficult to find functional differences based on their morphological differences [15]. To overcome these problems, we proposed a method for predicting the formation of MDSCs by comprehensively indexing the differentiation and maturation of myeloid lineage cells [17]. Various indices and effector molecules have been reported to define multiple MDSC populations [1]. While utilizing all these indices can present a valuable approach, it is confronted with the inherent complexity that cannot be fully addressed [18,19]. In fact, effector molecules produced by neutrophils, such as reactive oxygen species or neutrophil extracellular traps, exert dual effects, influencing both anti-tumor activity and immunosuppression [1,11]. In prior studies, we have defined CD33^+^CD16^hi^ as a neutrophil-like cell subset, encompassing both typical neutrophils and various MDSC subsets [17]. In this context, we proposed the utilization of GPI-80 coefficient variation (CV) within the CD33^+^CD16^hi^ cell subset as a comprehensive method to assess the variation of MDSCs [17]. This method serves as an approach to address the complexity that cannot be fully encapsulated by attempting to “describe everything”. Moreover, it offers the convenience of rapid analysis of limited blood samples and simultaneous measurements of CD33^hi^ monocyte-like cell subsets within the same sample.

In this study, we investigated the relationship between the induction of anti-tumor neutrophils and MDSCs by observing the time course of neutrophils during BCG treatment using this method. The findings of this study will help evaluate the state of anti-tumor immunity induced via BCG.

## 2. Materials and Methods

### 2.1. Human Peripheral Blood and Urine Collection

This study included 14 patients who were diagnosed with NMIBC at the Department of Urology, Yamagata University Hospital, between April 2017 and January 2019. All methods were performed in accordance with the relevant guidelines and regulations. All volunteers provided informed consent prior to blood and urine collection. This study was approved by the Ethics Committee of Yamagata University, Faculty of Medicine (approval number: 2019-311). Clinicopathological descriptions of the patients are presented in Table 1. Blood (10 mL) and urine (30–50 mL) samples were immediately collected before BCG infusion therapy. Appropriate treatments were provided to each patient based on their medical condition.

### 2.2. Flow Cytometric Analysis

Human whole blood cells were stained with antibodies, as described previously [17]. Briefly, whole blood was aliquoted into microtubes (50 μL of blood per tube) and incubated with Fc blocker (BioLegend, San Diego, CA, USA) for 5 min. After blocking the Fc receptors, whole blood was incubated with each antibody for 30 min at 4 °C and then treated with pre-warmed BD Phosflow Lyse/Fix buffer (1 mL; BD Biosciences, San Jose, CA, USA) for 10 min at 37 °C to lyze the red blood cells and fix the white blood cells. The urine cells (100 μL of 0.5 to 5 × 10^5^ cells per tube) were incubated with Fc blocker after washing with phosphate-buffered saline (PBS) and reacted with each antibody for 30 min at 4 °C. After re-washing with PBS, the cells were fixed with 2% formaldehyde in PBS. Mouse whole blood cells were collected from the heart, as previously described [20]. Peritoneal exudate cells (PECs) were collected by injecting RPMI 1640 containing 10% heat-inactivated fetal calf serum (FCS; Biowest, Nuaillé, France) and 10 U/mL low-molecular weight heparin (Mochida Pharmaceutical, Tokyo, Japan). For intracellular staining, the cells were fixed and permeabilized using a BD Cytofix/Cytoperm™ Fixation/Permeabilization kit (BD Biosciences), according to the manufacturer’s instructions. Mouse leukocytes were stained with antibodies, as described previously [20]. The cells were analyzed via flow cytometry using a FACSCanto II (BD Biosciences). The mean fluorescence intensity (MFI) and robust coefficient of variation (CV) were determined using FlowJo software version 7.6.5 (TreeStar, Ashland, OR, USA).

### 2.3. Antibodies

The antibodies used in this study were as follows: fluorescein isothiocyanate (FITC)-anti-human CD14 mAb (MφP9), phycoerythrin (PE)-anti-human CD16 mAb (3G8), and allophycocyanin (APC)-anti-human human leukocyte antigen DR isotype (HLA-DR) mAb (G46-6) from BD Biosciences; FITC-anti-human CD15 mAb (HI98), APC- or FITC-anti-human CD16 mAb (3G8), brilliant violet 421-anti-human CD33 mAb (WM53), PE-anti-human CD163 mAb (GHI/61), PE-anti-human CD197 mAb (G043H7), PE-anti-human C-X-C motif chemokine ligand 10 (CXCL10) (J034D6), APC-anti-mouse I-A/I-E mAb (M5/114.15.2), FITC-anti-mouse CD45 mAb (30-F11), PerCP-Cy5.5-anti-mouse Ly6C mAb (HK1.4), brilliant violet 421-anti-mouse Ly6G mAb (1A8), brilliant violet 421-anti-mouse Gr-1 mAb (RB6-8C5), and PE-streptavidin from BioLegend; APC-anti latency-associated peptide-1 (LAP; the N-terminal region of transforming growth factor-β1 precursor) mAb (#27232) and biotin-anti-mouse CXCL10 goat Ab (#BAF466) from R&D systems (Minneapolis, MN, USA); and PE-anti-human GPI-80 mAb (3H9) from MBL (Nagoya, Japan). For isotype-matched control mAbs, fluorescent substance-conjugated mouse IgG1 (MOPC-21), mouse IgG2a controls (G155-178), and rat IgG2a (RTK2758) were obtained from BD Biosciences and BioLegend.

### 2.4. Comprehensive Analysis of RNA

Polymorphonuclear leukocytes were purified from peripheral blood using Ficoll–Paque (GE Healthcare Life Sciences, Pittsburgh, PA, USA), as described previously [21]. Cells from blood and urine samples were prepared from the same patients after the 6th BCG infusion therapy (*n* = 3; median age, 70; age range, 55–83 years; male subjects, 3; pTis, 1; pT1, 2; and EAU high-risk group, 3). Total RNA was purified from cells using TRIzol (Thermo Fisher Scientific, Waltham, MA, USA), according to the manufacturer’s instructions. Each RNA sample was reverse transcribed and amplified twice using an Amino Allyl MessageAmp™ II RNA Amplification kit (Life technologies, Carlsbad, CA, USA). A comprehensive analysis of mRNA using a 3D-Gene^®^ DNA tip microarray was performed by Kamakura Techno-Science (TORAY Group, Kamakura, Japan). Gene ontology analysis was performed using laboratory life system information (Tohoku Chemical, Hirosaki, Japan).

### 2.5. Mouse Abdominal Tumor Model

C57BL/6J mice were purchased from CLEA Japan (Tokyo, Japan). The mice were maintained under specific pathogen-free conditions at the Yamagata University Research Center for Molecular Genetics and the Yamagata University Animal Center. Food (CE-2, CLEA Japan) and water were provided ad libitum. All mice were handled in accordance with the guidelines of the Animal Care and Use Committee of Yamagata University. The mice used in these experiments were 10–13 weeks of age (body weight > 23 g). This study was approved by the Ethics Committee for Animal Experiments at Yamagata University (approval numbers R2146 and R3083). The B16F10 murine melanoma cell line was obtained from Prof. Naoto Ishii (Department of Immunology, Tohoku University, Miyagi, Japan). These cells were cultured in RPMI 1640 (Thermo Fisher Scientific) and supplemented with 10% FCS, 5 mM of L-glutamine, 50 U/mL of penicillin G potassium, and 50 µg/mL of streptomycin sulfate. B16F10 cells (5 × 10^4^ cells/100 μL/head) were implanted into the peritoneal cavity at 0 d. BCG (Immunobladder^®^, Japan strain; Nippon Kayaku, Tokyo, Japan; 0.4 mg/mL) was stocked in an antibiotic mixture (10 mg/mL of streptomycin sulfate and 10,000 U/mL of penicillin) at −80 °C. BCG diluted 10-fold with PBS (40 μg/100 μL/head) or vehicle (antibiotic mixture, 100 μL/head) was injected into the peritoneal cavity at 0 and 7 d. Ultra-LEAF™ (low endotoxin and azide-free) antibodies (anti-Ly6G mAb, 1A8; control rat IgG2a, RTK2758; 100 μg/50 μL/head; BioLegend) were intravenously injected into the tail vein at 0 and 7 d. The endpoint of the abdominal tumor model was evaluated according to a previous study [22]. Since mice die within 1 d when the tumor score reaches 5–6, mice with a score of 5 were euthanized via over-anesthesia, in accordance with animal welfare ethics.

### 2.6. Statistical Analysis

All data were presented as scattered dots and means. Statistical analyses were performed using Prism software version 5.03 (GraphPad Software, San Diego, CA, USA), as indicated in each figure legend. *p*-values of <0.05 were considered significant. Principal component analysis (PCA) was performed using EZR version 1.35 (Saitama Medical Center, Jichi Medical University, Saitama, Japan), which is a graphical interface for R (R Foundation for Statistical Computing, Vienna, Austria); it is a modified version of the R commander [23].

## 3. Results

### 3.1. BCG Therapy Did Not Induce MDSC Production in Peripheral Blood

**Figure 1:**
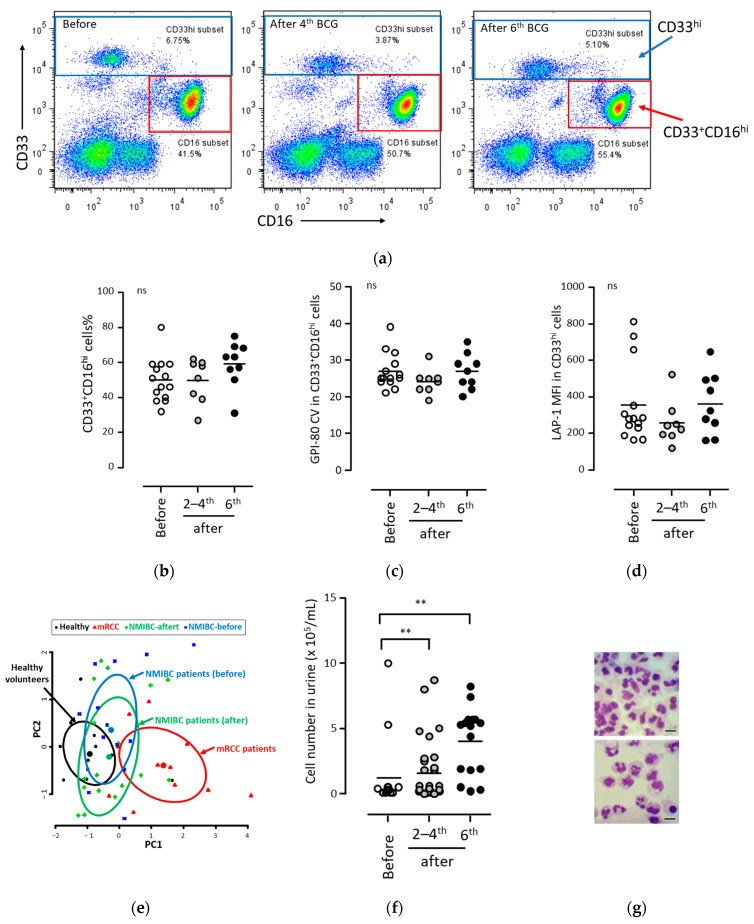

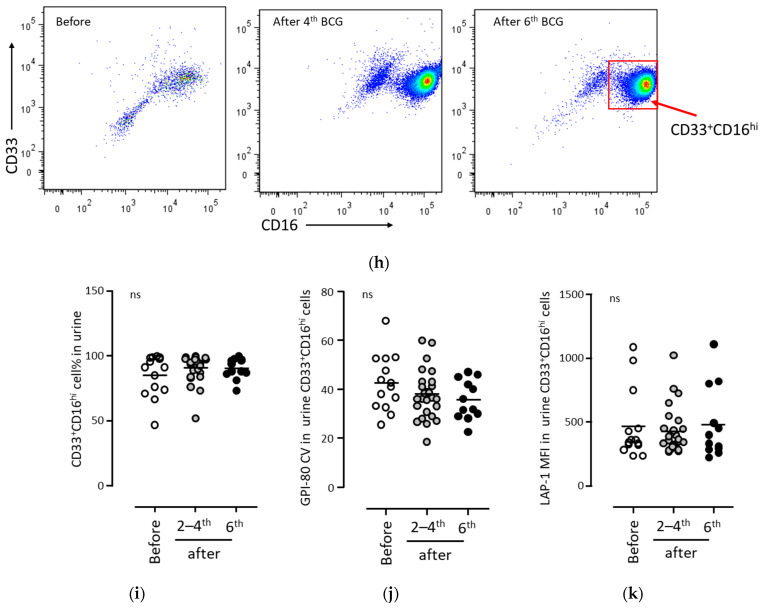
Comparison of MDSC indices in peripheral blood and urine during BCG infusion therapy. (**a**) Representative data of peripheral blood cells obtained from the bladder of patients and analyzed using flow cytometry. Blood was collected before BCG infusion therapy (**left panel**), after one week from the 4th BCG infusion (**middle panel**), and after one week from the 6th BCG infusion (**right panel**). (**b**–**d**) Comparison of MDSC indices in peripheral blood during BCG infusion therapy. The production indices of MDSCs, (**b**) CD33^+^CD16^hi^%, (**c**) GPI-80 CV in CD33^+^CD16^hi^ cells, and (**d**) LAP-1 MFI in CD33^hi^ cells were analyzed using peripheral blood samples. The blood was collected before BCG infusion therapy (before, open circle), after one week from the 2nd to 4th BCG infusions (2–4th, gray circle), and after one week from the 6th BCG infusion (6th, closed circle). (**e**) Differences in the MDSC indices of myeloid cells (neutrophils and monocytes) in peripheral blood obtained from patients with mRCC and patients with NMIBC using PCA. Blood from healthy volunteers, patients with mRCC, and patients with NMIBC (before BCG infusion, after 1 week from the 2nd to 6th BCG infusions) were collected, and then myeloid cells (CD33^+^CD16^hi^ cells and CD33^hi^ cells) in the peripheral blood were analyzed using flow cytometry, as shown in Figure 1a. The parameters CD16^hi^%, GPI-80 CV, and LAP-1 MFI were used for PCA. Each ellipse indicates 50% concentration of each group, and each closed symbol shows the center of the ellipse. (**f**) Cell concentration in urine samples collected from the patients. (**g**) Representative microscopic observations of the cells present in urine after BCG infusion therapy. The cells present in urine were collected from two patients with NMIBC after the 6th BCG infusion and were stained with May–Giemsa. Bar, 10 μm. (**h**) Representative data of effusion cells present in urine collected from the bladder of patients and analyzed using flow cytometry. Urine was collected before BCG infusion therapy (**left panel**), after one week from the 4th BCG infusion (**middle panel**), and after one week from the 6th BCG infusion (**right panel**). (**i**–**k**) Comparison of MDSC indices in urine during BCG infusion therapy. Urine was collected before BCG infusion therapy (before, open circle), after one week from the 2nd to 4th BCG infusions (2–4th, gray circle), and after one week from the 6th BCG infusion (6th, closed circle). The indices of MDSCs, (**i**) CD33^+^CD16^hi^%, (**j**) GPI-80 CV in CD33^+^CD16^hi^ cells, and (**k**) LAP-1 MFI in CD33^+^CD16^hi^ cells were analyzed. Statistical analyses were performed using the Kruskal–Wallis test with Dunn’s post-hoc test. Each bar is presented as the mean of data (** *p* < 0.01). Abbreviations: ns, not significant; MDSC, myeloid-derived suppressor cell; BCG, *Bacillus Calmette–Guérin*; NMIBC, non-muscle-invasive bladder cancer; mRCC, metastatic renal cell cancer; and PCA, principal component analysis.

We first investigated whether the BCG treatment induced the production of MDSCs, as described in a previous study [17]. Peripheral blood was collected from patients with NMIBC before and after BCG therapy, after which the blood cells were analyzed using flow cytometry (Figure 1a). The gating strategies of flow cytometry are shown in Figure S1a–c. The blood samples after BCG therapy consisted of blood collected after the second to fourth BCG infusions (2–4th) and the sixth BCG infusion (6th). The main indices for MDSC production, percentage, and GPI-80 CV of the CD33^+^CD16^hi^ cell population and the LAP1 MFI of the CD33^hi^ cell population were measured (Figure 1b–d). Peripheral blood samples were difficult to obtain from all patients in all treatments; therefore, a related multi-group test was not possible. However, a sufficient number of specimens was obtained for statistical evaluation. There were no significant differences in CD33^+^CD16^hi^%, GPI-80 CV, and LAP1 MFI before and after BCG therapy (Figure 1b–d). The indices of patients with NMIBC before and after BCG therapy were compared with those of patients with metastatic renal cell carcinoma (mRCC) using PCA, which revealed that the indices of patients with NMIBC were similar to those of the healthy controls (Figure 1e). These results indicated that multiple infusions of BCG does not induce MDSC production in the peripheral blood.

### 3.2. BCG Therapy Did Not Induce MDSC Production in Urine

Next, neutrophils present in the urine were analyzed. The concentration of cells in the urine significantly increased after BCG treatment compared to that before BCG treatment (Figure 1f). The cells in urine were quantified via flow cytometry, and their morphology was determined using microscopy. The cells in urine were neutrophilic in morphology (Figure 1g). A typical example of flow cytometric analysis is shown in Figure 1h. The gating strategies of flow cytometry are shown in Figure S1e–g. Most cells were identified as CD33^+^CD16^hi^ after repeated BCG treatments (Figure 1i).

Previously, it was reported that CD14^+^ monocyte subsets were detected in urine during BCG therapy [24]. In this study, we also investigated the expression of CD14 and HLA-DR as monocyte markers (Figure S3). However, unfortunately, the detection of CD14^+^ cells in urine was significantly more challenging when compared to CD16^+^ cells. Additionally, the presence of HLA-DR^+^ cells via cell surface staining was less than 10%. Furthermore, we examined the expression of CD197 and CD163 as markers for M1 and M2 macrophages, respectively, but both CD197^+^ or CD163^+^ cells were less than 5% (Figure S3c–f). Based on these observations, it was considered that, with our measurement sensitivity, neutrophils predominate among the white blood cells in urine. Another possibility to consider is that this issue may be related more to technical considerations than to biological phenomena.

In order to clarify whether the cells in urine showed characteristics of MDSCs, the indices of MDSCs were analyzed. There was no increase in the indices of MDSCs (GPI-80-CV and LAP-1) after BCG treatment compared to those before BCG treatment (Figure 1j,k). These results indicated that multiple infusions of BCG may not induce MDSC production in the urine.

### 3.3. Intracellular Expression of CXCL10 and HLA-DR Was Enhanced in Human Urinary Neutrophils during BCG Treatment

The activities of anti-tumor neutrophils and MDSCs are considered to be part of the activated neutrophil function, and their activities are mainly present in tumor microenvironments or inflamed tissues, but not in the peripheral blood [2,3]. It has been previously established that neutrophils in peripheral blood and infiltrated tissues exhibit distinct characteristics [25], and comparing neutrophils from the peripheral blood and infiltrated tissues is considered valuable for exploring neutrophil diversity [26]. In this study, the adjustment of neutrophil-derived mRNA from the peripheral blood and urine samples was efficiently performed without the need for enzymatic processing, and this adjustment was conducted simultaneously in a short timeframe. To the best of our knowledge, there have been no reported investigations conducted with such a methodology. Therefore, we deemed that by extrapolating neutrophil function in urine post-BCG infusion, we could distinctly detect changes related to anti-tumor effects.

**Figure 2:**
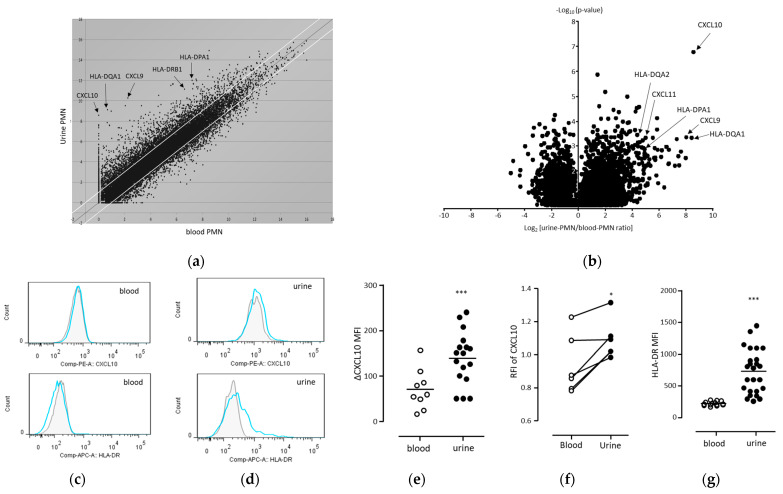
Upregulation of CXCL10 and MHC class II in human neutrophils in urine during BCG infusion therapy. (**a**,**b**) Comprehensive analysis of mRNA expression in urine-derived neutrophils compared to peripheral blood neutrophils. Blood and urine were collected from three patients after one week from the 6th BCG infusion. Comprehensive analysis of mRNA in neutrophils was performed using a DNA tip microarray. (**a**) Cluster analysis after adjustment and standardization. The mRNA expression in neutrophils obtained from urine samples (vertical axis) or peripheral blood (horizontal axis) was analyzed. White lines indicate the thresholds for genes that are upregulated or downregulated > 2-fold between urine- and blood-derived neutrophils. A relatively higher expression in urine-derived neutrophils is indicated using arrows, including expression for CXCR3 ligands (CXCL9 and CXCL10) and MHC class II (HLA-DRB1, HLA-DPA1, and HLA-DQA1). (**b**) Volcano plot depicting the differentially expressed genes between peripheral blood-derived and urine-derived neutrophils after the 6th BCG infusion. The horizontal axis denotes the fold change in mRNA expression in neutrophils from the urine and blood, while the vertical axis represents the –log_10_ (*p*-value) for a *t*-test of differences in neutrophils from the blood and urine. These data represent the top 6000 genes of the –log_10_ (*p*-value). The gene expressions of CXCR3 ligands (CXCL9, CXCL10, and CXCL11) and MHC class II (HLA-DQA2, HLA-DPA1, and HLA-DQA1) were also detected as characteristic features of urine-derived neutrophils (arrows). (**c**,**d**) Representative data of intracellular-stained neutrophilic cells obtained via flow cytometric analysis. The CD33^+^CD15^+^ neutrophilic cells in the blood (**c**) or urine (**d**) samples were obtained from the same patient who was treated with 4th BCG infusions and are presented as CXCL10 MFI (**upper panels**) and HLA-DR MFI (**lower panels**). Gray-closed histograms indicate each background staining, and light blue line histograms denote the staining of CXCL10 or HLA-DR. (**e**–**g**) Comparison of intracellular expression of (**e**,**f**) CXCL10 and (**g**) HLA-DR in neutrophilic cells from the blood (open circle) and urine (closed circle) samples. These samples were collected after one week from the 2nd to the 6th BCG infusions (after each infusion). (**e**) ΔCXCL10 MFI was calculated as follows: ΔCXCL10 MFI = (MFI of PE-conjugated anti-CXCL10 mAb staining) − (MFI of PE-conjugated control IgG staining). (**f**) The neutrophilic cells from patients who underwent their 5th BCG infusion or more, and from whom both peripheral blood and urine samples could be collected simultaneously, were analyzed. The samples with a cell viability in urine of less than 90% were excluded due to elevated non-specific fluorescence. The RFI of CXCL10 was calculated as follows: RFI of CXCL10 MFI = (MFI of PE-conjugated anti-CXCL10 mAb staining)/(MFI of PE-conjugated control IgG staining). (**e**,**g**) Each bar is presented as the mean of data. Statistical analyses were performed using the Mann–Whitney test (*** *p* < 0.001). (**f**) Statistical analyses were performed using the Wilcoxon signed-rank test (* *p* < 0.05; *n* = 6). Abbreviations: CXCL9, chemokine (C-X-C motif) ligand 9; HLA-DRB1, major histocompatibility complex, class II, DR β1; HLA-DPA1, major histocompatibility complex, class II, DP α1; HLA-DQA1, major histocompatibility complex, class II, DQ α1. BCG, *Bacillus Calmette–Guérin*; CXCL10, chemokine (C-X-C motif) ligand 10; HLA-DR, major histocompatibility complex class II cell surface receptor; MFI, mean fluorescence intensity; and RFI, relative fluorescence intensity.

In the cluster analysis, urine-derived neutrophils exhibited an increased expression of a group of major histocompatibility complex (MHC) class II genes and a series of chemokines, CXCL9 and CXCL10, whose receptor was CXCR3 (Figure 2a). Volcano analysis also confirmed the upregulation of a group of MHC class II genes and a series of chemokines, CXCL9, CXCL10, and CXCL11, whose receptor was CXCR3 (Figure 2b).

In previous studies, the effects of BCG on neutrophils were well known to result in the increased expression of TRAIL (TNFSF10), GRO-α, MIP-1α (CCL3), MIF, and CXCL8 [10,12]. In this study, we compared peripheral blood and urine (Table 2 and data from Figure S1). As a result, we observed less than a two-fold increase in the expression of TRAIL. Furthermore, there was no significant increase in the levels of GRO-α, MIP-1α (CCL3), MIF, and CXCL8. On the other hand, the CXCL10 and MHC class II antigens showed an increase of more than four-fold in expression, and similar chemokines, CXCL9 (MIG) and CXCL11 (I-TAC), which are induced by IFN-γ, also exhibited an increase of more than eight-fold in expression (Table 2). Therefore, in this study, we focused on the CXCL10 and MHC class II antigens, which had the highest rate of increase.

The upregulation of CXCL10 and MHC class II proteins was confirmed based on protein expression levels by performing flow cytometric analysis via intracellular antibody staining using clinical samples (Figure 2c–g). The analysis was performed as shown in Figure S2a–i. The expression levels of CXCL10 and HLA-DR in urine-derived neutrophils (CD33^+^CD15^+^ cells) were significantly higher than those in peripheral blood neutrophils.

The urine samples exhibited a strong non-specific FITC fluorescence, and there was a considerable variability in non-specific fluorescence between samples. Therefore, to account for the potential influence of non-specific FITC fluorescence on PE-CXCL10 expression, we calculated the relative fluorescence intensity (RFI). As a result, we observed an enhancement of CXCL10 expression in neutrophils in the urine of patients who underwent their fifth or more BCG infusions and in samples where enough peripheral blood and urine could be measured simultaneously (Figure 2f). These results indicate that intracellular CXCL10 and HLA-DR were upregulated in urine-derived neutrophils following BCG treatment.

**Figure 3:**
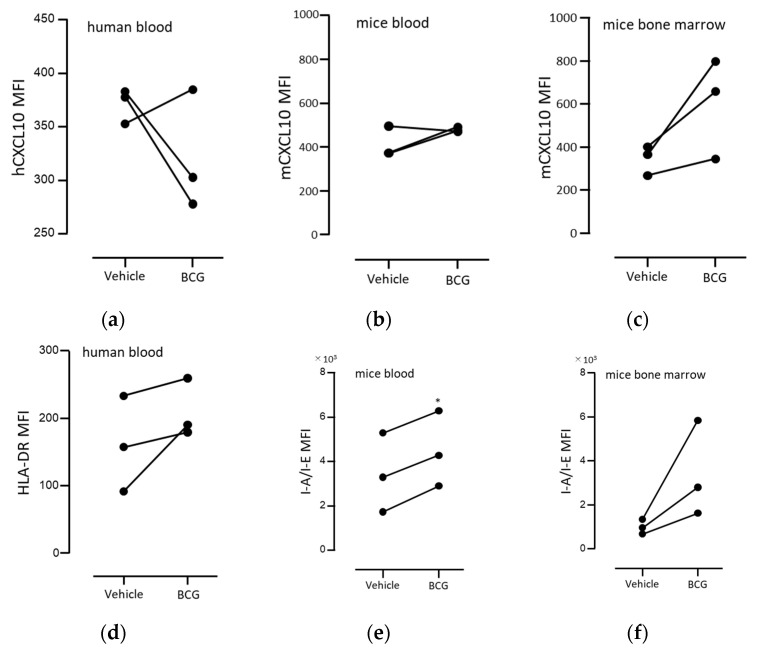
Effect of BCG on CXCL10 and MHC-II expression in human or mice neutrophilic cells in vitro. Human (**a**,**d**) or mouse (**b**,**e**) peripheral blood was diluted ten-fold in 10% FCS RPMI1640, or mouse bone marrow cells (4 × 10^6^/mL; **c**,**f**) were incubated with or without 4 μg/mL of BCG for 20 h. Following incubation, the expression levels of CXCL10 (**a**–**c**) and MHC class II (**d**–**f**) in human (CD33^+^CD15^+^) or mouse neutrophils (CD45^+^Ly6G^+^) were analyzed, as described in Figure S2. Statistical significance was calculated with the paired *t*-test, * *p* < 0.05 (*n* = 3). Abbreviations: BCG, *Bacillus Calmette–Guérin*; CXCL10, chemokine (C-X-C motif) ligand 10; HLA-DR, human major histocompatibility complex class II cell surface receptor; MFI, mean fluorescence intensity; and I-A/I-E, mouse major histocompatibility complex class II cell surface receptor.

Interestingly, previous studies have reported that BCG stimulation in vitro does not induce mRNA upregulation of CXCL10 in neutrophils [27,28]. In fact, we attempted BCG stimulation in vitro using human peripheral blood, but we could not detect an increase in CXCL10 production in both human and mouse peripheral blood (Figure 3a,b). However, when we stimulated mouse bone marrow cells, we observed a tendency for neutrophils to produce CXCL10 (Figure 3c). Based on these findings, it was considered that the conditions for detecting CXCL10-producing neutrophils may be rare and be observed under certain conditions with immature neutrophils. On the other hand, the expression of MHC class II in peripheral neutrophils was observed to be induced even upon in vitro BCG stimulation (Figure 3d–f).

### 3.4. BCG Administration Induced the Intracellular Expression of CXCL10 and HLA-DR in Mouse Peritoneal Exudate Neutrophils

**Figure 4:**
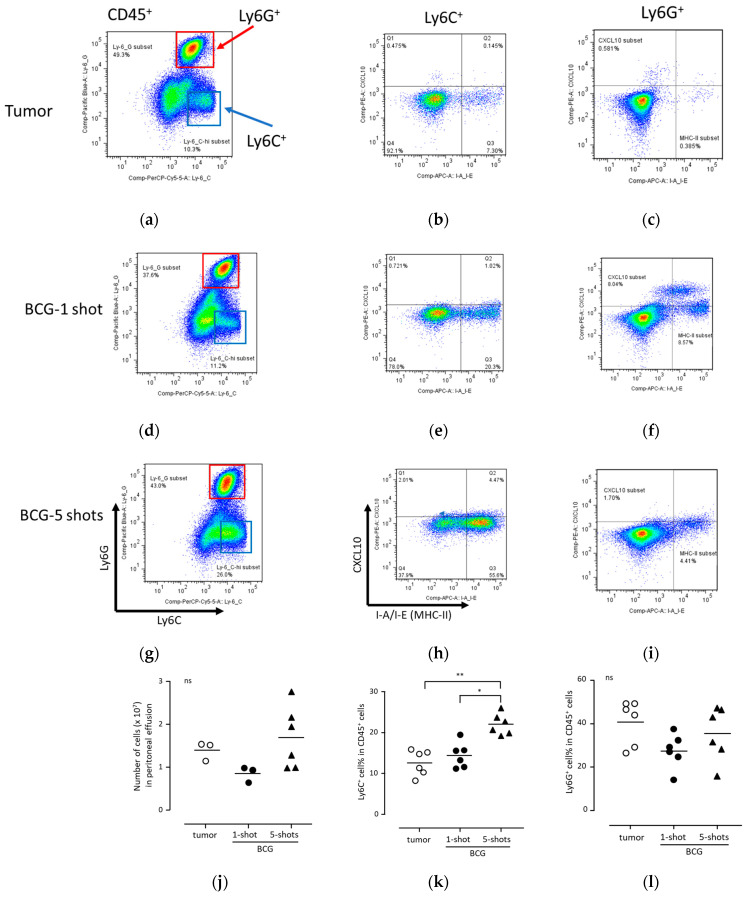

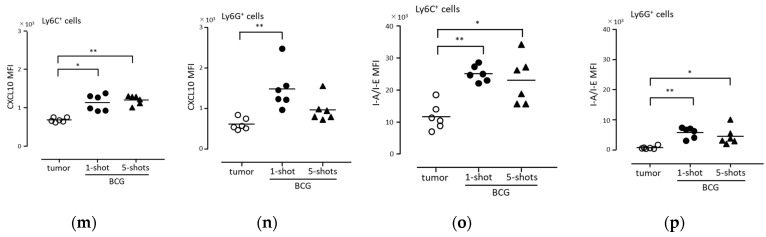
Upregulation of CXCL10 and MHC class II in monocytes and neutrophils in peritoneal effusion cells after BCG injections. Mice were injected with B16F10 cells (5 × 10^4^ cells/100 μL/head), and the PECs were collected after two weeks. The PECs induced after one injection of BCG (40 μg/head) after 16 h and the PECs induced after five repeated injections of BCG (40 μg/head) after 16 h from the final injection are presented as “1-shot” and “5-shots”, respectively. These PECs were intracellularly stained with each antibody, and the relative expression (MFI) of CXCL10 and I-A/I-E was analyzed in CD45^+^Ly6C^+^ cells and CD45^+^Ly6G^+^ cells, respectively. (**a**–**i**) Representative flow cytometric analysis of mouse monocytes (Ly6C^+^ cells) and neutrophils (Ly6G^+^ cells) via flow cytometry. The (**a**–**c**) panels present flow cytometric analysis of the PECs induced 2 weeks after B16F10 cell injection (presented as “Tumor”). The (**d**–**f**) panels show representative flow cytometric analysis of the PECs induced 16 h after the administration of BCG (presented as “1-shot). The (**g**–**i**) panels indicate representative flow cytometric analyses of the PECs induced via five repeated BCG injections at one-week intervals. The PECs were collected 16 h after the final BCG administration (presented as “5-shots”). The left panels (**a**,**d**,**g**) show CD45^+^ leukocytes presented with the gates of Ly6C^+^ cells (monocytic cells) and Ly6G^+^ cells (neutrophilic cells). (**j**–**l**) The number and proportion of myeloid cells (Ly6C^+^ and Ly6G^+^ cells) of the PECs. The peritoneal effusion cells obtained after injection of B16F10 cells are presented as “tumor” (open circles). The cells induced 16 h after a single administration of BCG are presented in the group “1-shot” (closed circles). The cells induced via five repeated injections of BCG are presented in the group “5-shots” (closed triangles). The (**j**) number of the cells in peritoneal fluid were counted using a hemocytometer, and the proportions of (**k**) Ly6C^+^ cells and (**l**) Ly6G^+^ cells in CD45^+^ leukocytes were analyzed via flow cytometry. (**m**–**p**) The intracellular expression levels of CXCL10 and MHC-II (I-A/I-E) in mouse monocytes (Ly6C^+^ cells) and neutrophils (Ly6G^+^ cells) after BCG injection. These PECs were intracellularly stained with each antibody, and the relative expression (MFI) of (**m**,**n**) CXCL10 and (**o**,**p**) I-A/I-E was analyzed in (**m**,**o**) CD45^+^Ly6C^+^ cells and (**n**,**p**) CD45^+^Ly6G^+^ cells, respectively. Statistical analyses were performed using the Kruskal–Wallis test with the Dunn’s post-hoc test. Each bar is presented as the mean of data. * *p* < 0.05; ** *p* < 0.01; and ns, not significant. Abbreviations: PECs, peritoneal exudate cells; CXCL10, C-X-C motif chemokine ligand 10; BCG, *Bacillus Calmette–Guérin*; and MFI, mean fluorescence intensity.

In order to confirm that the upregulation of CXCL10 and MHC class II proteins in neutrophils was induced by BCG in vivo, we attempted to reproduce this in a mouse model. In the mouse model, BCG was intraperitoneally administered, and PECs were analyzed. As a control, mice were intraperitoneally administered with B16F10 melanoma cells, which induce MDSC formation. We utilized the B16F10 cell line, a commonly employed murine melanoma cell line in many cancer transplantation models. Interestingly, previous research papers have demonstrated a neutrophil-dependent anti-tumor effect with this cell line [29]. Therefore, in our study, we chose to employ the B16F10 cell line with the aim of obtaining results that could be applicable to various research endeavors. The gating strategies for the PECs are shown in Figure S2j–n, and representative patterns of CXCL10 and I-A/I-E (MHC class II) are shown in Figure 4a–i.

In the mouse PECs as well, similar to human neutrophils in urine, there were instances where negative and positive cell populations did not distinctly segregate. Additionally, we observed MHC-II-only positive cells and CXCL10-only positive cells, with a clear MHC-II^+^CXCL10^+^ double-positive cell subset accounting for approximately 1–10% of the population, even following BCG administration. Therefore, we conducted an analysis of the mean fluorescence intensity (MFI) for the entire Ly6G^+^ cell subset.

No difference was observed in the number of leukocytes in the peritoneal cavity after a single administration of BCG (one shot) and after five weekly administrations (five shots; Figure 4j). In addition, there was no difference in the number of leukocytes after one shot and tumor cell transplantation (Figure 4j). Unlike human urine during BCG treatment, monocytes (Ly6C^+^) were detected in the peritoneal cavity of mice, and the proportion of monocytes in PECs significantly increased after five shots (Figure 4k). However, the proportion of neutrophils between the one-shot and five-shots groups did not differ (Figure 4l). These observations indicate that BCG administration induces the intraperitoneal effusion of both neutrophils and monocytes, and that repeated BCG administration enhances the intraperitoneal effusion of monocytes.

In monocytes, a significant increase in CXCL10 production was observed in the one-shot and five-shots groups, compared to that under tumor transplantation (Figure 4m). In neutrophils, the one-shot group showed significantly increased CXCL10 production, but not the five-shots group (Figure 4n). In contrast, I-A/I-E (MHC class II) expression was significantly increased in both monocytes and neutrophils in both the one-shot and five-shots groups, compared to that under tumor transplantation (Figure 4o,p). These results indicate that BCG administration induces both CXCL10 production and MHC class II expression in neutrophils after a single BCG administration in mice.

### 3.5. Anti-Tumor Immunity Was Elicited with Neutrophils Induced via BCG

**Figure 5:**
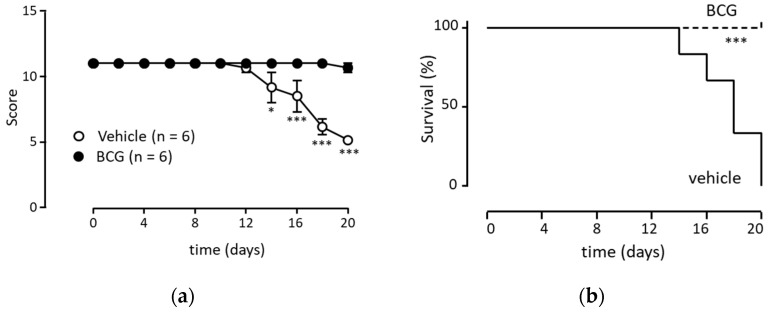

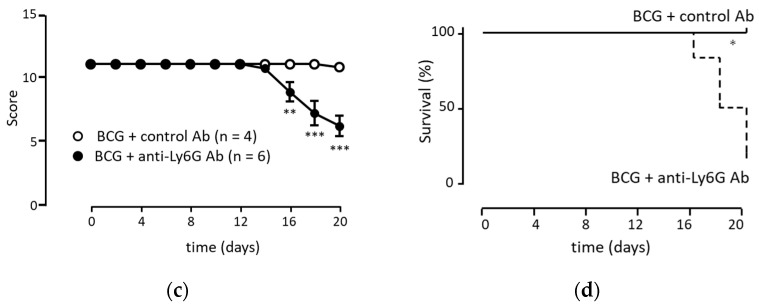
BCG enhances anti-tumor activity via Ly6G^+^ neutrophils. (**a**) Inhibition of melanoma proliferation via BCG injection. Melanoma cells (B16F10; 5 × 10^4^ cells/0.1 mL/head) were implanted into the peritoneal cavity at 0 d using male mice. BCG (40 μg/100 μL/head) or vehicle (penicillin–streptomycin cocktail, 100 μL/head) was also injected into the peritoneal cavity at 0 and 7 d. These data are presented as the mean ± standard deviation from three independent experiments (vehicle, *n* = 6; BCG, *n* = 6). When the mice presented a score of ≤5, the mice were euthanized and counted as “score 5” until 20 d. Statistical analyses were performed using the two-way ANOVA test, with the post-hoc test using Bonferroni correction (* *p* < 0.05; *** *p* < 0.001). (**c**) Suppression of BCG-mediated anti-tumor activity owing to the depletion of neutrophils. Melanoma cells were implanted into the peritoneal cavity at 0 d. BCG (40 μg/100 μL/head) was also injected into the peritoneal cavity at 0 and 7 d, the same as in (**a**). Furthermore, antibodies (100 μg/50 μL/head; control mAb or anti-Ly6G mAb) were injected into the tail vein at 0 and 7 d. These data are presented as the mean ± standard deviation from three independent experiments (vehicle, *n* = 4; BCG, *n* = 6). When the mice presented a score of ≤5, the mice were euthanized and counted as “score 5” until 20 d. Statistical analyses were performed using the two-way ANOVA test, with the post-hoc test using Bonferroni correction (** *p* < 0.01; *** *p* < 0.001). (**b**,**d**) present a survival curve generated using the data from (**a**,**c**) using the Kaplan–Meier method (a score ≤ 5 was considered to reflect death in mice). Statistical analyses were performed using the log-rank (Mantel–Cox) test (* *p* < 0.05; *** *p* < 0.001). BCG, *Bacillus Calmette–Guérin*.

Using a BCG-administered mouse model, we investigated whether BCG administration induced anti-tumor effects. BCG was administered when the tumor cells were transplanted into the peritoneal cavity and administered again one week later. While intraperitoneal tumor cell transplantation without BCG administration resulted in a score of five or less (mice with a score of five died within a day) within 20 d, BCG administration performed twice almost suppressed the decrease in score (Figure 5a); BCG administration protected all mice (Figure 5b).

**Figure 6:**
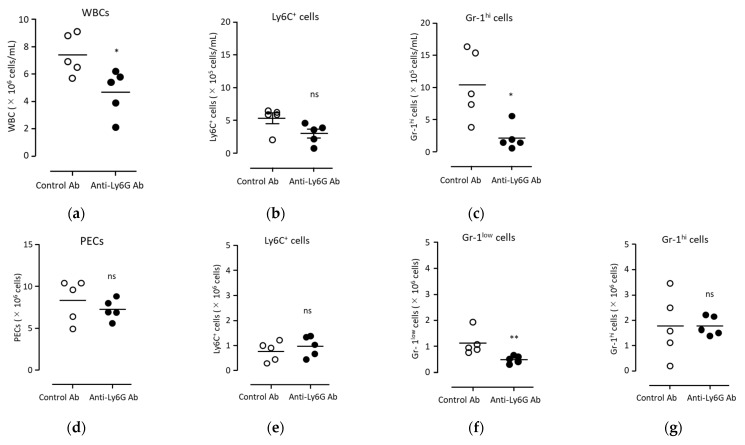
The number of myeloid cells (Ly6C^+^ and Gr-1^hi^ cells) in the peripheral blood and PECs after injections of anti-Ly6G mAb. After 16 h from the administration of BCG (40 μg/100 μL/head) into the peritoneal cavity and the injection of antibodies (100 μg/50 μL/head; rat IgG2a, open circle; or anti-Ly6G mAb, closed circle) into the tail vein, the peripheral blood and peritoneal exudate were collected as described Section 2. WBCs in the (**a**) blood or (**d**) PECs were stained with Turk’s solution and counted using a hemocytometer. The proportion of (**b**) Ly6C^+^ cells and (**c**) Gr-1^hi^ cells in blood were measured via flow cytometry. The proportion of (**e**) Ly6C^+^ cells, (**f**) Gr-1low, and (**g**) Gr-1^hi^ cells in PECs was also measured. Each proportion of cell population was used for the calculation of cell concentration and number. Statistical analyses were performed using the Mann–Whitney test (* *p* < 0.05; ** *p* < 0.01; ns, non-significant). Each bar is presented as the mean of data from three independent experiments. PECs, peritoneal exudate cells; WBCs, white blood cells; BCG, Bacillus Calmette–Guérin.

**Figure 7:**
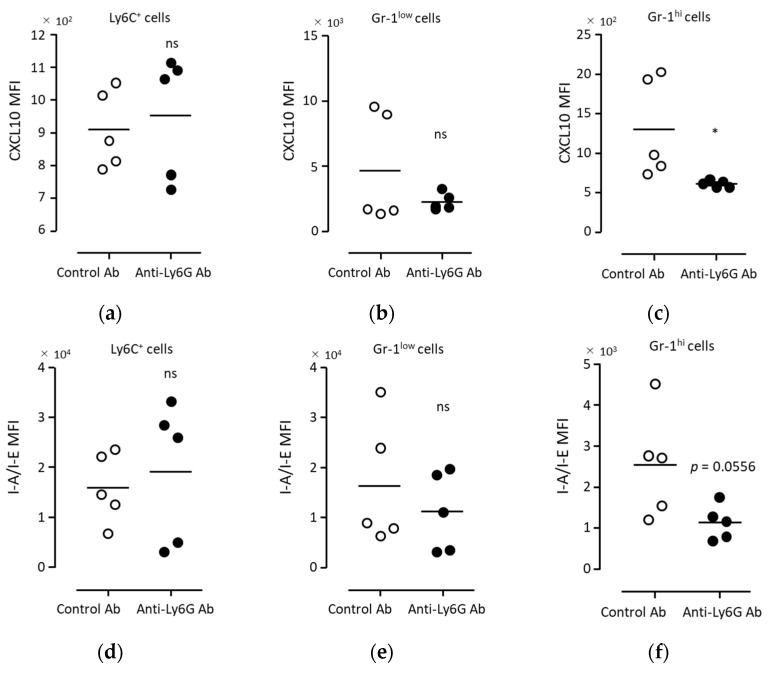
CXCL10 and MHC class II expression in neutrophils induced via BCG was inhibited via partial neutrophil depletion using anti-Ly6G mAbs. BCG (40 μg/100 μL/head) was injected into the peritoneal cavity, following which the antibodies (100 μg/50 μL/head; control mAb, open circle; or anti-Ly6G mAb, closed circle) were injected into the tail vein. Sixteen hours post-injection, PECs were collected and analyzed via flow cytometry. The relative expression levels (MFI) of CXCL10 (**a**–**c**) and I-A/I-E (**d**–**f**) were analyzed in CD45^+^Ly6C^+^ cells (**a**,**d**), CD45^+^Gr-1^low^ cells (**b**,**e**), and CD45^+^Gr-1^hi^ cells (**c**,**f**). Statistical analyses were performed using the Mann–Whitney test (* *p* < 0.05; ns, not significant). Each bar represents the mean of the data from three independent experiments. Abbreviations: BCG, *Bacillus Calmette–Guérin*; CXCL10, chemokine (C-X-C motif) ligand 10; MFI, mean fluorescence intensity; and PECs, peritoneal exudate cells.

In order to clarify whether the anti-tumor effect of BCG administration was due to neutrophils, a neutrophil-depleting antibody (anti-Ly6G mAb, 1A8) was administered to mice. Following antibody administration into the peripheral blood, the neutrophils were partially depleted (Figure 6a–c). To evaluate the neutrophil populations, another anti-neutrophil antibody, anti-Gr-1 mAb (RB6-8C5), was used after the administration of anti-Ly6G mAb. Anti-Gr-1 mAb binds to both Ly6C^+^ and Ly6G^+^ cells. Thus, we analyzed the Gr-1^hi^ cell population as neutrophils, separately from the Gr-1^low^ cell population (Figure S4). Gr-1^hi^ neutrophils were significantly depleted in the peripheral blood following anti-Ly6G Ab administration compared with that obtained after control Ab administration (Figure 6c). In contrast, in PECs, the number of Gr-1^low^ cells was significantly decreased after anti-Ly6G mAb administration, whereas that of Gr-1^hi^ neutrophils was not significantly decreased (Figure 6d–g). Under these conditions of partial neutrophil depletion, the anti-tumor effect of BCG administration was significantly inhibited (Figure 5c,d). Furthermore, in Gr-1^hi^ neutrophils, the expression level of CXCL10 was significantly decreased via anti-Ly6G mAb administration (Figure 7c). A trend of decreased MHC-II expression in neutrophils following Ly6G mAb administration was observed, although it did not reach statistical significance due to the considerable variability in the control group (Figure 7f). The number of Gr-1^hi^ cells was higher than that of monocytes and Gr-1^low^ cells among PECs (Figure 6d–g). Further, downregulation of CXCL10 and MHC-II via anti-Ly6G mAbs was not observed in monocytes and Gr-1^low^ cells (Figure 7a,d). These results indicate that CXCL10 production and MHC-II elevation in Gr-1^hi^ neutrophils were associated with the anti-tumor effects induced via BCG.

## 4. Discussion

This study demonstrated that the inflammatory response to BCG treatment in bladder cancer did not induce MDSC production. Furthermore, the formation of CXCL10- and MHC-II-expressing neutrophils was induced at the site of BCG injection in both humans and mice. Partial neutrophil depletion in peripheral blood could suppress the neutrophil and anti-tumor responses induced via BCG. These observations indicated that CXCL10 and MHC-II are upregulated in a specific subpopulation of neutrophils, which may be involved in inducing anti-tumor responses.

In the present study, CXCL10 production was observed in neutrophils induced via BCG administration in vivo. Although stimulation with BCG alone in vitro does not induce CXCL10 production in neutrophils [27], neutrophils can produce CXCL10 upon combined stimulation with interferon-γ and lipopolysaccharides (LPSs) or tumor necrosis factor-α [30]. The production of CXCL10 in urine during BCG treatment has been previously confirmed [31], and CXCL10 has been shown to play a key role in eliciting anti-tumor immune reactions [32]. Therefore, CXCL10 induced via BCG administration is considered important for anti-tumor immune reactions.

In this study, we measured intracellular chemokine CXCL10 production. Typically, to measure intracellular cytokines, Brefeldin A is used to inhibit secretion; however, we analyzed samples fixed from in vivo collections. Therefore, we were unable to achieve highly sensitive detection of accumulated CXCL10 production. Consequently, when conducting positive cell subset analysis via flow cytometry, we observed the presence of both MHC-II-only positive cells and CXCL10-only positive cells, indicating a heterogeneous population. While our representative data showed clear subsets of CXCL10-producing cells, there were instances where distinct subsets of CXCL10-producing cells were not observed. In fact, when utilizing mice in our study, CXCL10-positive cell subsets were more detectable 16 h after a single BCG administration, but their detection became challenging after five BCG administrations. It remains unclear whether this reduction in detectability was due to decreased secretion or hyperstimulation, rendering them unobservable after 16 h. Further investigations are required to understand the relationship between the number of BCG administrations and the timing of PEC collection. However, when we compared the mean fluorescence intensity (MFI) of the entire Ly6G^+^ cell subset, we observed a similar trend of increased CXCL10 and MHC-II expression, consistent with the results observed in the human urine samples.

The induction of MHC class II expression in neutrophils has been previously reported [16]. Recently, it was demonstrated that MHC class II-expressing neutrophils, known as neutrophil-derived antigen-presenting cells, induce anti-tumor responses via cross-presentation [15]. Therefore, MHC class II-expressing neutrophils induced via BCG treatment may be involved in enhancing the anti-tumor response.

The markers for MDSCs are similar to neutrophil activation markers [1,2,3,6]. In addition, the mechanism of suppressing T cell proliferation is related to the production of reactive oxygen species, which similarly suppresses proliferation in tumor cells [1,33]. Therefore, it is difficult to evaluate MDSCs, as neutrophils and monocytes during BCG therapy are already in an activated state [12,27]. In this study, we used a combination of the ratio of neutrophil-like cells, the coefficient of variation of GPI-80, and the expression level of LAP, to investigate MDSCs in the peripheral blood and urine during BCG therapy. This method revealed, for the first time, that BCG therapy does not induce MDSCs in the peripheral blood and urine. However, it remains uncertain whether our method can be universally applied to all MDSC subsets. The quantification of MDSCs presents inherent complexity and challenges in functional assessments [18,19]. Further investigations are warranted to address these issues.

Neutrophil anti-tumor activity and MDSC activity are two facets of the same coin. Although it was already known that neutrophils are involved in the anti-tumor activity induced via BCG administration [12], the kind of neutrophils involved remained unknown. This study showed that neutrophils involved in their anti-tumor activity involve a cell subpopulation with upregulated CXCL10 and MHC-II expression levels. In the future, if we can clarify the mechanism of this anti-tumor neutrophil induction, various immunotherapies using anti-tumor neutrophils or MDSCs will become possible. However, the large genetic difference between human and mouse neutrophils makes it difficult to apply the mouse model to human neutrophils. In fact, there is no homologous gene for *VNN2/GPI-80* in mice [34], and there is no homologous gene for *Ly6g* in humans [35]. In the future, the ability to evaluate anti-tumor neutrophils and MDSCs using common molecules, such as CXCL10 and MHC-II, in humans and mice will pave the way for the development of immunotherapy using mouse models.

In this study, since it was difficult to collect the cells exuding into the mouse bladder, we used an intraperitoneal tumor transplantation model. Neutrophil-like cells exuded in the human bladder and neutrophil-like cells exuded in the peritoneal cavity of mice after BCG administration were similar to anti-tumor neutrophils, and the anti-tumor response induced via BCG administration was dramatic in this intraperitoneal tumor implantation model. Interestingly, the anti-tumor effect that disappears with a neutralizing antibody against neutrophils (anti-Ly6G mAb) was associated with the decrease in the number of neutrophils in the peripheral blood, not the number of exuded cells into peritoneal cavity.

Furthermore, we observed an increase in CXCL10 and MHC-II in urine and peritoneal exudate cells (PECs), but this increase was not evident when stimulating peripheral blood from healthy individuals with BCG in vitro. However, during BCG administration, when we partially reduced peripheral blood neutrophils using anti-Ly6G mAbs, the rise in CXCL10 and MHC-II in PECs was suppressed. Anti-Ly6G mAbs administered into the peripheral blood are known to also act on neutrophils in the bone marrow [36]. From these findings, we deduced that the pre-changes leading to the increase in CXCL10 and MHC-II, occurring before their release as peripheral blood neutrophils, may take place within certain sites like the bone marrow during localized BCG administration. In the future, this mouse model will be useful for observing anti-tumor responses induced via BCG administration.

Many methods of neutrophil depletion have been reported [37]. LPS-induced mortality is enhanced via complete depletion of neutrophils with five sequential injections of anti-Ly6G mAb into the peritoneal cavity [38]. This mortality is also augmented by complete neutrophil depletion via the injection of diphtheria toxin in *PMN^DTR^* mice [38]. While arthritis does not develop in neutropenic mice (*Lyz2^Cre/Cre^Mcl1^flox/flox^* mice) [39], each method of controlling neutrophils induces a different phenomenon, indicating the ambiguity of the neutrophil population. In this study, the anti-tumor activity induced via BCG administration disappeared after partial neutrophil depletion, which was induced via a week’s interval injection of anti-Ly6G mAb into the tail vein. Previous studies have reported on the effects of administering anti-Ly6G mAb into the peripheral blood [36,40]. According to these reports, the administration of anti-Ly6G mAb into the peripheral blood does not alter the neutrophil migratory capacity, and the administration of the equivalent of 100 μg of anti-Ly6G antibodies results in neutrophil depletion within one hour. Therefore, the co-administration of anti-Ly6G mAb via the tail vein during intraperitoneal BCG administration has been presumed to remove pre-change neutrophils before the induction of CXCL10 and MHC-II. However, the responsiveness of MHC-II^+^CXCL10^+^ neutrophils to anti-Ly6G mAbs remains uncertain. This injection method may be an effective way to reveal the dynamic equilibrium and transient neutrophil functions that cannot be detected via the constant depletion of neutrophils. It is necessary to examine the route and the timing of anti-Ly6G mAb injection for the investigation of neutrophil function in future research.

To accurately assess the function of human MDSCs within solid tumors, the experimental conditions for retrieving these MDSCs are crucial, but they are notoriously challenging to control [19]. On the other hand, peripheral blood is easily accessible as a clinical sample, facilitating the observation of temporal changes. Understanding the state of neutrophil-like cells in the peripheral blood (comprising classical neutrophils and a mixture of various MDSC subsets) would be highly beneficial [26]. However, it is in infiltrated tissues that MDSC function becomes more evident, potentially not manifesting their functionality clearly through pre-changes in the peripheral blood [26]. In the future, elucidating the pre-changes in peripheral blood neutrophil-like cells during the early stages of BCG administration may lead to the proposal of clinically useful indicators that can be contrasted with MDSCs exhibiting pre-changes in the peripheral blood.

## 5. Conclusions

The absence of an increase in GPI-80 CV in our detection methods led us to conclude that MDSC induction is not likely to occur with weekly BCG administrations. The anti-tumor neutrophils induced via BCG exhibited upregulated CXCL10 and MHC class II expression levels. The anti-tumor subpopulation of neutrophils in the tumor microenvironment is derived from neutrophils in the peripheral blood induced early in the inflammatory response. In the future, the evaluation of anti-tumor precursor neutrophils in the peripheral blood will facilitate the determination of appropriate systemic conditions to induce maximum immunotherapeutic effects.

## Figures and Tables

**Table 1:** Baseline demographic and clinical characteristics.

Classification	Number
Age in years, median (range)	67 (50–89)
Male	10
Female	4
pTis	6
pTa	4
pT1	4
Intermediate ^1^	2
High ^1^	11
Very high ^1^	1

^1^ EAU (European Association of Urology) risk group.

**Table 2:** Upregulation of CXCL10 and MHC class II genes in human neutrophilic cells in urine during BCG infusion therapy.

Gene Symbol	Description	Log_2_ Ratio ^1^	*p*-Value ^2^
CXCL10	C-X-C motif chemokine ligand 10	8.59	1.68 × 10^−7^
HLA-DQα1	Major histocompatibility complex, class II, DQ alpha 1	8.44	4.53 × 10^−4^
CXCL9	C-X-C motif chemokine ligand 9	8.04	4.48 × 10^−4^
CXCL11	C-X-C motif chemokine ligand 11	5.06	4.71 × 10^−4^
HLA-DPβ1	Major histocompatibility complex, class II, DP beta 1	4.96	1.34 × 10^−3^
HLA-DPα1	Major histocompatibility complex, class II, DP alpha 1	4.81	5.77 × 10^−4^
HLA-DRβ1	Major histocompatibility complex, class II, DR beta 1	4.50	3.02 × 10^−3^
HLA-DQA2	Major histocompatibility complex, class II, DQ alpha 2	4.40	3.50 × 10^−4^
TNFSF10 (TRAIL)	Tumor necrosis factor superfamily member 10	1.04	3.49 × 10^−1^

^1^ Calculated value of “Log_2_ global normalization” difference between urine PMNs and peripheral blood PMNs. ^2^ Results of performing an unpaired *t*-test on comparison between urine PMNs and peripheral blood PMNs. PMNs, polymorphonuclear leukocytes.

## Data Availability

The data presented in this study are available from the corresponding author upon request.

## Supplementary Materials

The following supporting information can be downloaded at: https://www.mdpi.com/article/10.3390/biomedicines11113062/s1, Supplementary Figure S1: Representative gating strategy of flow cytometric analysis for cell surface staining samples; Supplementary Figure S2: Representative gating strategy of flow cytometric analysis for intracellular staining samples; Supplementary Figure S3: Representative flow cytometric analysis of urine monocytes and macrophage staining samples on the cell surface; Supplementary Figure S4: Representative flow cytometric analysis of mouse myeloid cells (Ly6C^+^ cells, Gr-1^low^ cells, and Gr-1^hi^ cells) in intracellular peripheral blood and PECs; Data S1: DNA array data.
