# Supplementary material for: Intracellular Major Histocompatibility Complex Class II and C-X-C Motif Chemokine Ligand 10-Expressing Neutrophils Indicate the State of Anti-Tumor Activity Induced by Bacillus Calmette–Guérin
Source: Biomedicines. 2023 Nov 15;11(11):3062. doi: 10.3390/biomedicines11113062 (PMC10669614; doi:10.3390/biomedicines11113062)
Supplement: Supplementary file 1 [file biomedicines-11-03062-s001.zip › Revised Supplementary_materials.pdf]

Supplementary materials

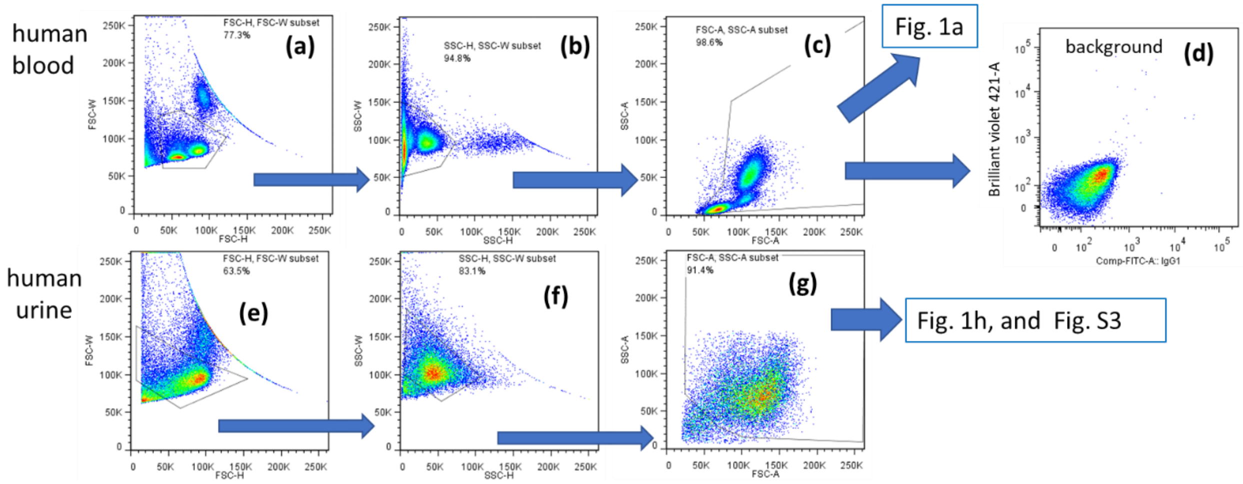

Supplementary Figure S1. Representative gating strategy of flow cytometric analysis for cell surface staining samples.

(a–d) Representative gating strategy for cell surface staining of peripheral blood cells. The panels for (a) FSC-H vs. FSC-W and (b) SSC-H vs. SSC-W show the gates for the exclusion of aggregated cells. The (c) FSC-A vs. SSC-A panel indicates the gate for the exclusion of cell debris. Panel (d) shows representative background staining for Brilliant violet 421 and FITC.

(e–g) Representative gating strategy for cell surface staining using urine samples. The panels for (e) FSC-H vs. FSC-W and (f) SSC-H vs. SSC-W show the gates for the exclusion of aggregated cells. The (g) FSC-A vs. SSC-A panel indicates the gate for the exclusion of cell debris. The background staining is shown in Fig. S3.

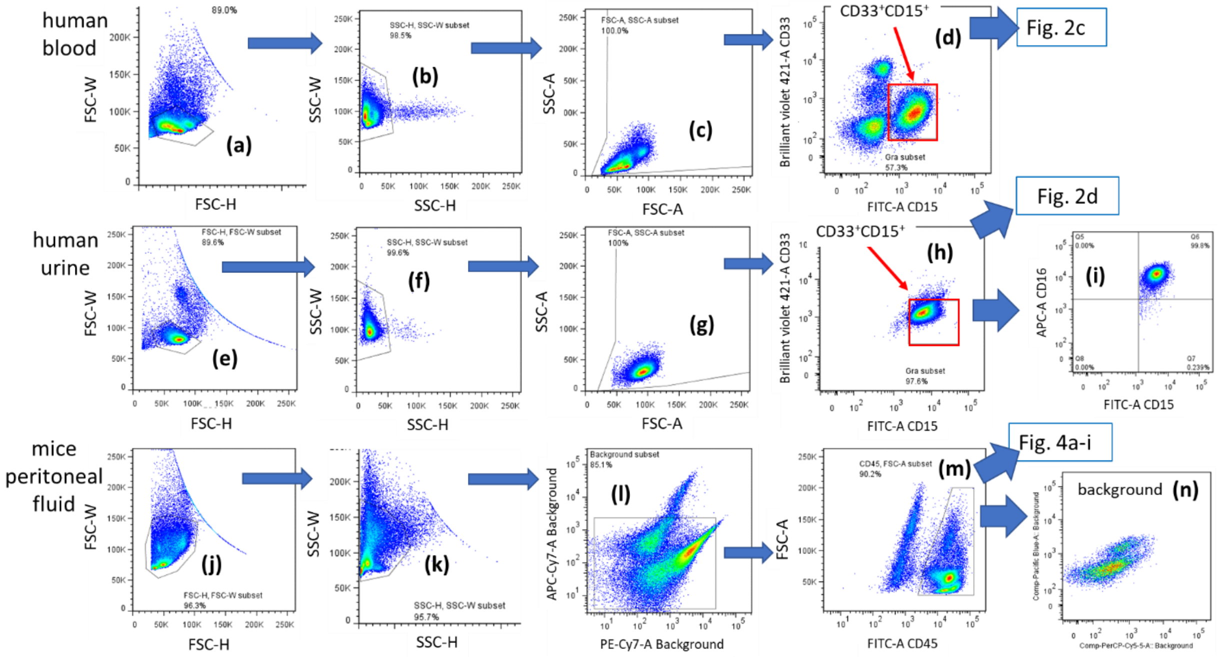

Supplementary Figure S2. Representative gating strategy of flow cytometric analysis for intracellular staining samples.

(a–d) Representative gating strategy for intracellular staining of peripheral blood cells. The panels for (a) FSC-H vs. FSC-W and (b) SSC-H vs. SSC-W show the gates for the exclusion of aggregated cells. The (c) FSC-A vs. SSC-A panel indicates the gate for the exclusion of cell debris. The (d) panel for FITC-CD15 vs. Pacific Blue-CD33 shows the gate for neutrophilic cells in the peripheral blood. The background staining is shown in Fig. 2c.

(e–i) Representative gating strategy for intracellular staining using urine samples. The panels for (e) FSC-H vs. FSC-W and (f) SSC-H vs. SSC-W show the gates for the exclusion of aggregated cells. The (g) FSC-A vs. SSC-A panel indicates the gate for the exclusion of cell debris. The panel of (e) FITC-CD15 vs. Brilliant violet 421-CD33 shows the gate for neutrophilic cells present in the urine. Panel (i) indicates that $CD15^{+}$ cells in urine are $CD16^{+}$ cells. The background staining is shown in Fig. 2d.

(j–n) Representative gating strategy for intracellular staining of mouse peritoneal fluid. The panels for (j) FSC-H vs. FSC-W and (k) SSC-H vs. SSC-W show the gates for the exclusion of aggregated cells. The panel for (l) PE-Cy7-A vs. APC-Cy7-A indicates the gate for excluding multiple self-fluorescence events from cell debris. The panel for (m) FITC-CD45 vs. FSC-A shows the gate for leukocytes in peritoneal fluid. Panel (n) shows representative background staining for Brilliant violet 421 and PerCP-Cy5.5. Brilliant violet 421-conjugated mAb and PerCP-Cy5.5-conjugated mAbs were reacted with the cells after the treatment with fixation and permeabilization.

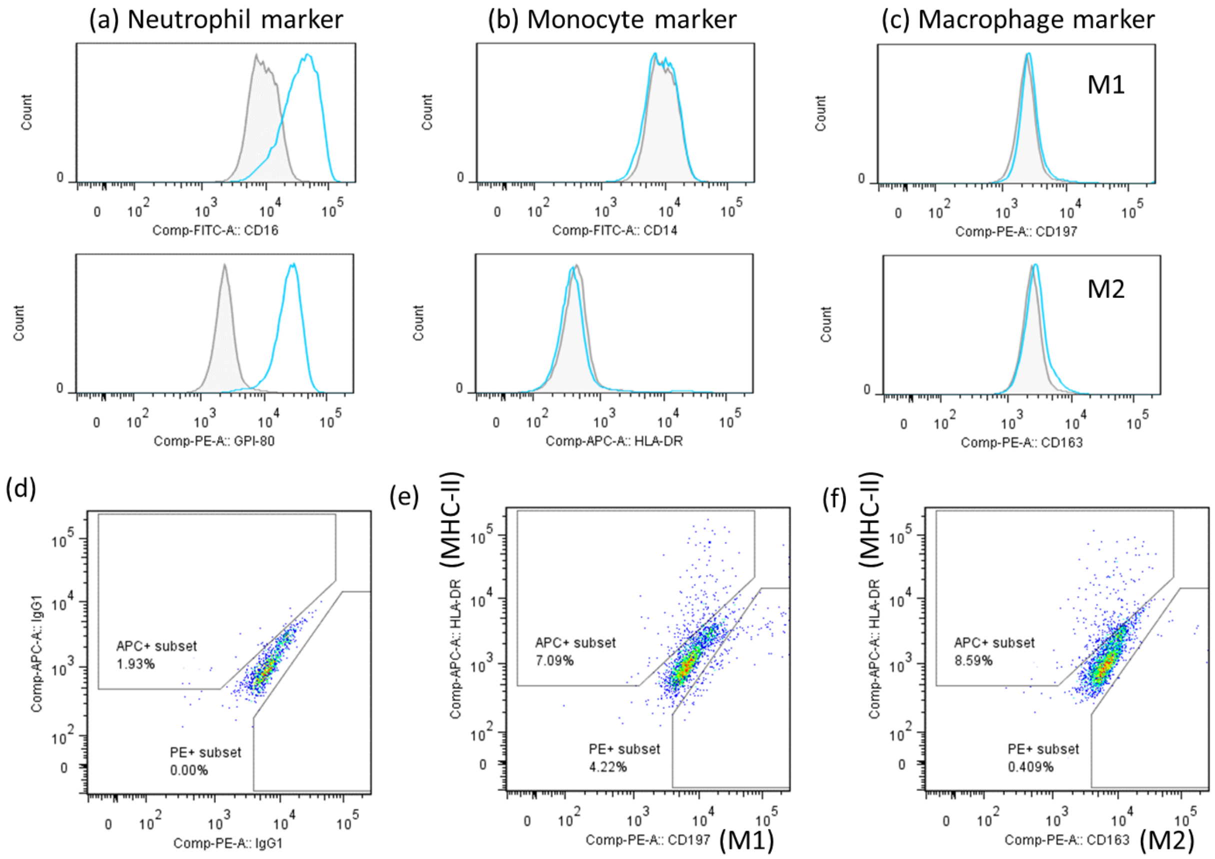

Supplementary Figure S3. Representative flow cytometric analysis of urine monocytes and macrophage staining samples on cell surface.

Urine samples were obtained from patients with NMIBC after 1 week from 2nd (a–c) and 5th BCG infusion (d–f). Each gating strategy was performed as described in Figure S1e–g (cell surface staining). (a–c) Representative histogram analysis of neutrophil, monocyte, and macrophage markers in urine sample. The gray-filled histograms in each panel indicate negative control mAbs staining. The light blue-line histogram of FITC-CD16 (upper panel) or PE-GPI-80 (lower panel) show as neutrophil marker (a), FITC-CD14 (upper panel) or APC-HLA-DR (lower panel) are monocyte marker (b), and PE-CD197 (upper panel) or PE-CD163 (lower panel) are as macrophage maker (c). (d–f) Representative dot plot analysis of macrophage markers. The panel for PE-IgG1 vs. APC-IgG1 shows as negative staining (d), PE-CD197 vs. APC-HLA-DR is M1 macrophages (e), and PE-CD163 vs. APC-HLA-DR is M2 macrophages (f). GPI-80^{+} cells in the sample (d–f) was 92.8%.

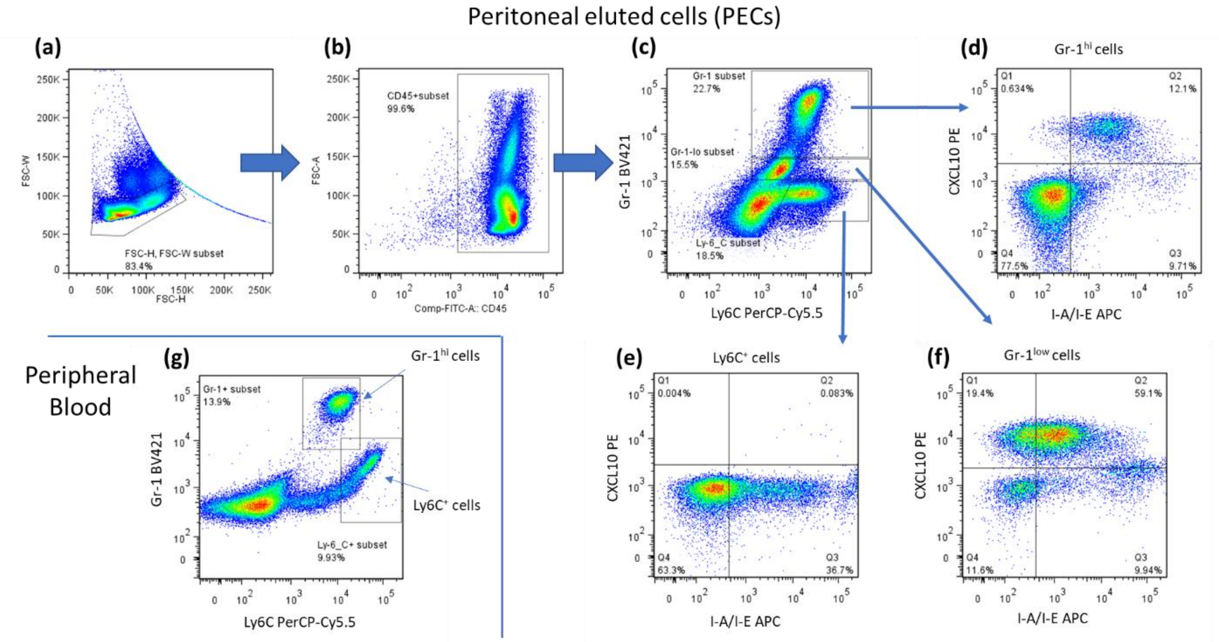

Supplementary Figure S4. Representative flow cytometric analysis of mouse myeloid cells (Ly6C^{+} cells, Gr-1^{low} cells, and Gr-1^{hi} cells) in intracellular peripheral blood and PECs.

After 16 h from intraperitoneal injection of BCG (40 μg/100 μL/head), PECs and peripheral blood cells were analyzed using flow cytometry. (a–f) present the gating strategy for the intracellular staining of PECs. The panels for (a) FSC-H vs FSC-W and (b) CD45 vs FSC-A show the gate for exclusion of aggregated cells and selection of leukocytes. The panel for (c) Ly6C vs Gr-1 indicates the gate for Ly6C^{+} cells, Gr-1^{low} cells, and Gr-1^{hi} cells. Representative analysis patterns of (d) Ly6C^{+} cells, (e) Gr-1^{low} cells, and (f) Gr-1^{hi} cells are shown as I-A/I-E (MHC class II) vs CXCL10. (g) Gating strategy for Ly6C^{+} cells and Gr-1^{hi} cells in peripheral blood cells. In the peripheral blood, there were no Gr-1^{low} cells. PECs, peritoneal exudate cells; BCG, *Bacillus Calmette-Guérin*; CXCL10, C-X-C motif chemokine ligand 10.
